# Differential effects of a combination of *Hibiscus sabdariffa* and *Lippia citriodora* polyphenols in overweight/obese subjects: A randomized controlled trial

**DOI:** 10.1038/s41598-019-39159-5

**Published:** 2019-02-28

**Authors:** María Herranz-López, Mariló Olivares-Vicente, Marina Boix-Castejón, Nuria Caturla, Enrique Roche, Vicente Micol

**Affiliations:** 1 Instituto de Investigación, Desarrollo e Innovación en Biotecnología Sanitaria de Elche (IDiBE) e Instituto de Biología Molecular y Celular (IBMC), Miguel Hernández University (UMH), Elche, 03202 Alicante, Spain; 2 Institute of Bioengineering and Department of Applied Biology-Nutrition, University Miguel Hernandez, Alicante Institute for Health and Biomedical Research (ISABIAL-FISABIO Foundation), Alicante, Spain; 3 Monteloeder S.L., Alicante, Spain; 4 CIBER, Fisiopatología de la Obesidad y la Nutrición, CIBERobn, Instituto de Salud Carlos III (CB12/03/30038), 07122 Palma de Mallorca, Spain

## Abstract

Plant-polyphenols have shown the capacity to ameliorate obesity-induced metabolic disturbances, both in cell and animal models, where most therapeutic approaches have failed. On the basis of previous research, a dietary supplement containing 500 mg of a combination of polyphenolic extracts from *Lippia citriodora* L. and *Hibiscus sabdariffa* L. (LC-HS), in the context of an equilibrated isocaloric diet, was evaluated in a double blind, placebo-controlled and randomized trial in 56 obese/overweight subjects for two months. Compared to controls, the consumption of the LC-HS polyphenols showed significant improvements in body weight, abdominal circumference of overweight subjects (−6.79 ± 0.80 cm in overweight LC-HS group *vs* −1.85 ± 0.83 cm in controls, *p* < 0.001) and body fat % (−1.33 ± 0.15% in overweight LC-HS group *vs* −0.66 ± 0.17% in controls, *p* < 0.05). Heart rate and systolic blood pressure also presented significant improvements in overweight LC-HS participants. However, changes were more modest in obese subjects. Further, LC-HS extract significantly reduced lipid content and increased AMPK activity in a hypertrophied adipocyte cell model. Therefore, consumption of 500 mg/day of LC-HS extracts enriched in polyphenols for two months in the context of an isocaloric diet by overweight subjects decreased symptoms associated to obesity-related diseases. Modulation of fat metabolism in adipose tissue, probably mediated by AMPK activation, is proposed as a molecular target to be explored in future research.

## Introduction

Obesity has reached global epidemic proportions. Given the absence of effective drugs with no side-effects, improving our understanding of the relationship between diet and health may be a realistic alternative. Obesity is associated with several metabolic abnormalities including insulin resistance, endothelial dysfunction and dyslipidemia, which are involved in the development of type 2 diabetes, hypertension and cardiovascular diseases. Very often, these changes start to be evident during the overweight phase (Body Mass Index <30). While there has been considerable progress in understanding the molecular mechanisms underlying metabolic disorders, their successful treatment remains limited^1,2^.

The causal link between excessive fat storage in obesity and metabolic disturbances is mediated by altered metabolic homeostasis in adipose tissue. Excessive accumulation of triglycerides leading to adipocyte hypertrophy compromises cell function, initiating an inflammatory process related to oxidative stress, which can lead to metabolic disorders associated with obesity^3,4^. Among the putative targets to ameliorate metabolic diseases, the energy sensor AMP-activated protein kinase (AMPK) has been proposed as an important therapeutic target for obesity^5–7^. Its activation leads to activation of catabolic pathways such as lipolysis and fatty acid oxidation and to inhibition of anabolic pathways such as lipogenesis and gluconeogenesis^8^.

Our previous research has accumulated enough evidence and has identified several herbal extracts with the capacity to alleviate metabolic stress and to modulate different molecular and cellular targets in cell culture and animal models^9–12^. These extracts include *Hibiscus sabdariffa* (HS), *Lippia citriodora* (LC), *Olea europaea* leaf and extra virgin olive oil extracts^13^. Most of these effects were correlated with the activation of the energy sensor AMP-activated protein kinase (AMPK). Using an insulin resistant hypertrophic adipocyte model, we have seen that HS polyphenols decreased metabolic stress in glucotoxicity and/or lipotoxicity events through the modulation of pathways associated with energy management and inflammation^5,6^. Additionally, HS polyphenols exhibited the capacity to inhibit triglyceride accumulation, oxidative stress and the secretion of inflammatory adipokines that regulate the infiltration of non-resident macrophages to adipose tissue^3,9,14^. Moreover, the efficacy of HS polyphenolic extract has also been demonstrated in animal models by preventing hepatic steatosis in hyperlipidemic mice through the regulation of the expression of genes involved in glucose and lipid homeostasis^10^, and lowering blood pressure and improving endothelial function^11^. Bioavailability studies performed in rat model and adipocytes suggest that quercetin-3-O-β-D-glucuronide and its aglycone may be responsible for the observed effects^10,12,13,15^.

On the other hand, studies on LC, namely lemon verbena, polyphenols showed favorable effects such as decreased lipogenesis, enhanced fatty acid oxidation and activation of the AMPK pathway, probably through PPAR-gamma receptor activation and adiponectin^16^. Similar to the HS polyphenol extract, the continuous administration of LC polyphenolic extract prevented fatty liver disease (FLD) and improved lipid metabolism in hyperlipidemic animal model. Interestingly, the results on lipid and glucose metabolism obtained in the hyperlipidemic mice revealed the possibility that HS and LC reach similar as well as complementary targets^10,16^. These findings prompted us to explore the effects of a combination of HS and LC in obese mice fed a high-fat diet (HFD). A recent report has shown the capacity of this combination to decrease obesity and its complications, improving the metabolism of HFD mice through increased thermogenesis-inducing genes in the white adipose tissue, and correlating with increased phosphorylation of AMPK and fatty-acid oxidation in the liver^17^. Finally, a randomized controlled trial performed in overweight subjects has demonstrated that the combination of HS and LC can modulate appetite-related peptides, as well as reduced blood pressure when compared to placebo, helping probably to a better management of body weight in the context of an equilibrated isocaloric diet^18^.

Therefore, the objective of this study was to assess the comparative efficacy of the abovementioned formulation, containing both LC and HS extracts (LC-HS), in two groups of overweight/obese subjects under risk of developing metabolic syndrome to search for differential effects between obese and overweight subjects. The capacity of a dietary supplement containing this combination coupled with isocaloric diet to modulate anthropometric parameters, as well as to improve several metabolic and hematological parameters associated with metabolic syndrome, such as blood pressure and heart rate, was also differentially studied in overweight and/or obese participants. Then, we assayed the potential of this combination to activate the AMPK-enzyme and to reduce triglyceride accumulation in the hypertrophied adipocyte model.

## Results

### Intervention study results

#### Anthropometric parameters

**Figure 1:**
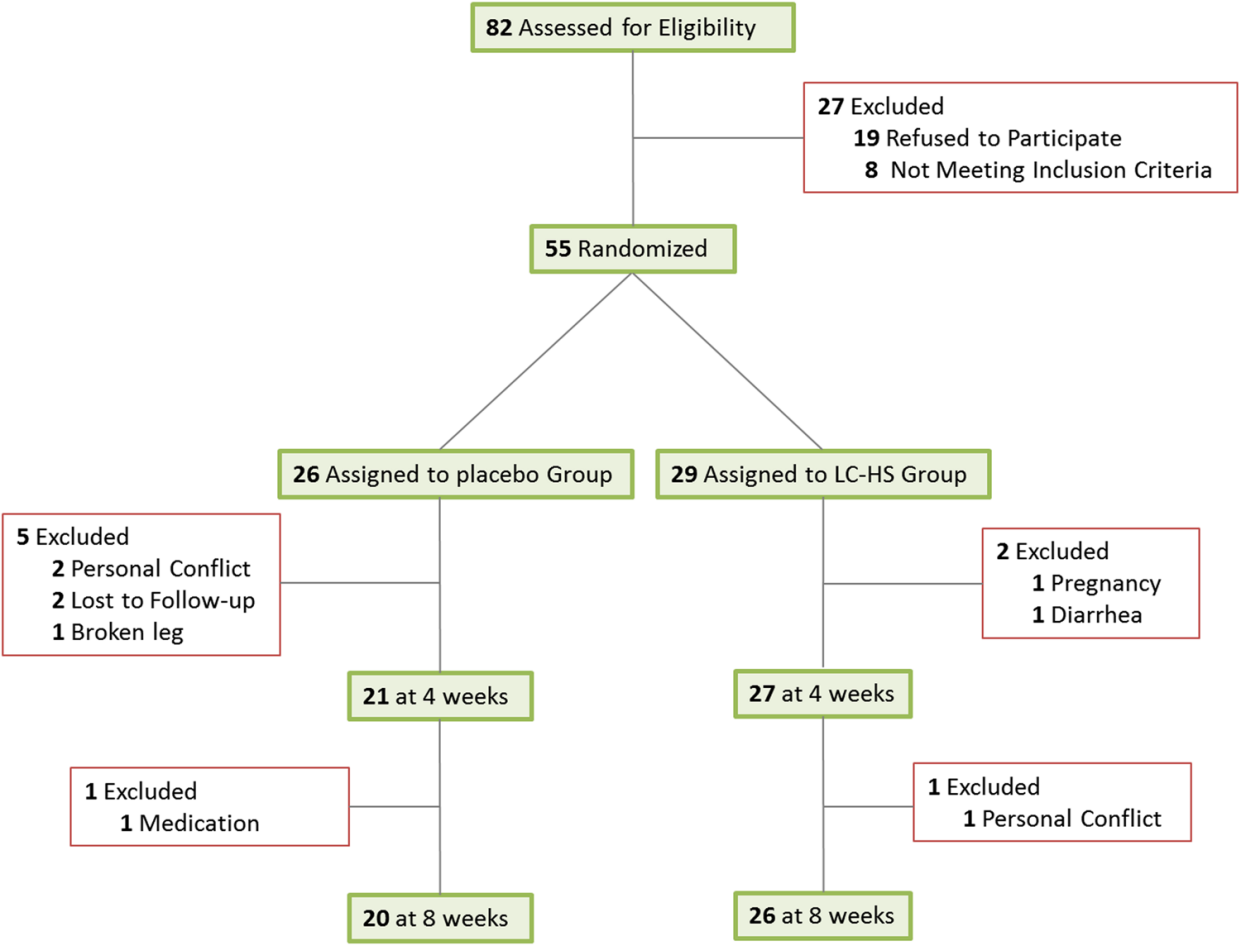
Study design and flow chart of the double blind, placebo controlled and randomized trial using LC-HS combination in 56 overweight volunteers.

Subjects in the study were sedentary lifestyle at baseline. Characteristics of the two overweight and the two obese groups were well matched and no significant differences were found at baseline (Table 1A,B). Study design and flow chart are shown in Fig. 1 (see suplementary file for full trial protocol). During the intervention study, participants were instructed to walk every day for 30 minutes as was reflected at weekly meetings. The results showed an overall improvement in the anthropometric parameters determined in the groups taking LC-HS compared to control after two months, particularly in body weight, abdominal circumference and percentage of body fat. Changes were more significant in the overweight group (Table 1A). The LC-HS groups exhibited a higher decrease of body weight compared to the control group, and significant differences were observed mainly between the control and LC-HS overweight groups (−1.96 ± 2.49 kg vs. −3.69 ± 0.34 kg, respectively, *p* < 0.05) and the control and LC-HS obese (−2.17 ± 0.95 kg vs. −4.68 ± 0.67 kg, respectively, *p* < 0.05) groups (Table 2). Both abdominal circumference parameters (ACs) were also reduced over the two months of treatment, particularly in overweight people consuming LC-HS (Table 1). After two months, only the AC1 presented a significantly reduction of −6.79 ± 0.80 cm in the overweight LC-HS group, compared to the −1.85 ± 0.83 cm of reduction in the overweight control group, *p* < 0.001 (Table 2A). Consistently with this data, % body fat (BF) decreased over time in overweight and obese groups (Table 1) but the overweight group consuming the extract lost significantly more body fat (−1.33 ± 0.15%) compared to the control group (−0.66 ± 0.17%, *p* < 0.05) after two months (Table 2A). In line with the above, a decrease in BMI was also observed in overweight and obese groups (Table 1), but the overweight LC-HS group exhibited a stronger decrease in BMI compared to the control, showing statistically significant differences (−1.46 ± 0.14 vs. −0.78 ± 0.28, respectively, *p* < 0.05) (Table 2A).

**Table 1:** Intragroup analysis of anthropometric, vital signs and biochemical measurements after one and two months of intervention.

A) OVERWEIGHT	LC-HS (N = 16)			Control (N = 10)		
	BASELINE	MONTH 1	MONTH 2	BASELINE	MONTH 1	MONTH 2
**Anthropometric parameters**						
Body weight	67.97 ± 8.42	65.48 ± 8.07****	64.28 ± 8.32****	67.83 ± 6.74	66.25 ± 7.28**	65.87 ± 7.60*
Body mass index (BMI) kg/m^2^	26.62 ± 1.85	25.64 ± 1.69****	25.16 ± 1.73****	27.31 ± 1.60	26.67 ± 1.88**	26.53 ± 2.25*
AC1 (cm)	93.91 ± 10.85	87.89 ± 9.00***	86.42 ± 9.71****	83.60 ± 6.32	81.75 ± 5.97**	81.75 ± 5.96*
AC2 (cm)	100.4 ± 10.50	96.58 ± 9.20**	94.61 ± 9.52****	95.69 ± 7.20	92.78 ± 6.79*	90.66 ± 5.92**
Triceps skinfold thickness (mm)	28.13 ± 9.02	25.13 ± 7.94**	23.92 ± 8.64***	27.29 ± 3.05	25.63 ± 2.31*	25.13 ± 2.16**
% Body fat (BF)	44.05 ± 2.89	43.16 ± 2.66****	42.72 ± 2.61****	42.87 ± 1.13	42.39 ± 1.15**	42.21 ± 1.28**
**Vital signs**						
Heart rate (BPM)	81.40 ± 9.64	75.57 ± 8.23***	72.87 ± 7.30****	74.75 ± 10.06	74.08 ± 13.57	73.50 ± 13.05
Systolic BP mm Hg	129.2 ± 14.84	118.2 ± 15.48****	108.6 ± 9.18****	120.8 ± 15.66	117.8 ± 15.11	115.4 ± 15.40
Diastolic BP mm Hg	79.60 ± 11.89	75.07 ± 10.44	68.53 ± 10.27****	74.75 ± 12.75	75.08 ± 6.68	69.33 ± 10.35
**Biochemical parameters**						
Glucose mg/dl	90.08 ± 12.35	86.17 ± 11.45	86.75 ± 8.67	92.82 ± 15.83	94.73 ± 11.96	94.73 ± 18.42
Triglycerides mg/dl	80.33 ± 57.87	74.17 ± 40.67	79.17 ± 40.91	77.18 ± 25.63	72.64 ± 20.01	75.73 ± 20.78
Total Cholesterol mg/dl	245.4 ± 25.69	219.8 ± 27.95***	211.8 ± 27.22***	223.7 ± 28.09	197.0 ± 16.61**	196.6 ± 14.53***
HDL mg/dl	61.81 ± 7.70	60.69 ± 7.62	60.69 ± 9.26	59.36 ± 8.00	58.09 ± 7.87	57.91 ± 7.67
LDL mg/dl	165.6 ± 23.70	146.1 ± 20.83***	133.2 ± 18.81****	148 0.8 ± 27.15	124.5 ± 18.19**	123.5 ± 14.04***
**B) OBESE**	**LC-HS (N** = **10)**			**Control (N** = **10)**		
	**BASELINE**	**MONTH 1**	**MONTH 2**	**BASELINE**	**MONTH 1**	**MONTH 2**
**Anthropometric parameters**						
Body weight	88.23 ± 11.57	86.09 ± 11.14*	83.55 ± 11.57***	86.16 ± 6.85	84.03 ± 6.53**	83.99 ± 6.42*
Body mass index (BMI) kg/m^2^	33.92 ± 4.93	33.07 ± 4.48*	32.06 ± 4.50**	34.62 ± 3.50	33.78 ± 3.59**	33.77 ± 3.63*
AC1 (cm)	88.80 ± 10.41	85.35 ± 9.91*	82.63 ± 9.80**	101.9 ± 13.21	100.4 ± 12.35*	98.61 ± 10.84
AC2 (cm)	103.7 ± 9.03	100.6 ± 7.69	97.86 ± 9.08***	112.3 ± 9.74	110.5 ± 10.04	106.6 ± 8.87*
Triceps skinfold thickness (mm)	32.14 ± 9.46	30.43 ± 7.55	28.86 ± 8.15**	34.38 ± 5.04	32.69 ± 4.32	32.0 ± 4.47
% Body fat (BF)	44.92 ± 2.97	44.23 ± 2.98**	43.63 ± 2.84**	47.35 ± 2.42	46.86 ± 2.56**	46.55 ± 2.23**
**Vital signs**						
Heart rate (BPM)	76.90 ± 10.20	72.85 ± 10.29**	68.90 ± 9.43****	77.25 ± 6.21	76.75 ± 9.35	78.50 ± 9.01
Systolic BP mm Hg	129.5 ± 15.44	118.1 ± 15.93**	111.1 ± 8.19***	135.5 ± 21.25	125.0 ± 20.91	122.0 ± 16.17
Diastolic BP mm Hg	75.60 ± 9.42	67.20 ± 7.64*	64.90 ± 5.71****	83.63 ± 12.55	77.63 ± 10.29	73.75 ± 13.56
**Biochemical parameters**						
Glucose mg/dl	100.1 ± 19.79	96.71 ± 15.82	95.14 ± 17.74	95.50 ± 8.32	93.13 ± 10.64	89.75 ± 8.57*
Triglycerides mg/dl	115.1 ± 68.99	76.86 ± 23.41	86.0 ± 37.66	97.25 ± 58.40	86.63 ± 36.16	106.6 ± 52.04
Total Cholesterol mg/dl	229.3 ± 27.46	213.7 ± 29.88*	208.7 ± 28.85**	236.5 ± 22.83	217.3 ± 30.34*	222.5 ± 36.90
HDL mg/dl	57.45 ± 7.12	55.73 ± 7.13*	55.09 ± 7.06**	58.50 ± 7.35	55.38 ± 10.77	56.0 ± 10.13
LDL mg/dl	146.5 ± 23.07	130.8 ± 23.58**	121.0 ± 22.80****	160.4 ± 21.23	144.5 ± 25.37*	145.9 ± 32.34

Intra-group statistical analysis at the endpoint compared to the baseline is reported as follows: *p < 0.05; **p < 0.01; ***p < 0.001; ****p < 0.0001. Data are expressed as the mean ± se.

**Table 2:** Intergroup analysis of changes in anthropometric, vital signs and biochemical measurements after one and two months of dietary supplement use.

A) OVERWEIGHT	Changes in the first month		Changes in two months	
	LC-HS (N = 16)	Control (N = 10)	LC-HS (N = 10)	Control (N = 10)
**Anthropometric parameters**				
Body weight	−2.49 ± 0.26	−1.58 ± 0.45	−3.69 ± 0.34*	−1.96 ± 2.49
Body mass index (BMI) kg/m^2^	−0.98 ± 0.10	−0.64 ± 0.17	−1.46 ± 0.14*	−0.78 ± 0.28
AC1 (cm)	−5.22 ± 0.94**	−1.85 ± 0.56	−6.79 ± 0.80***	−1.85 ± 0.83
AC2 (cm)	−3.82 ± 1.06	−2.92 ± 0.95	−5.79 ± 1.05	−5.03 ± 1.21
Triceps skinfold thickness (mm)	3.00 ± 0.75	1.67 ± 0.55	−4.21 ± 0.88	−2.16 ± 0.59
% Body fat (BF)	−0.89 ± 0.12*	−0.49 ± 0.13	−1.33 ± 0.15*	−0.66 ± 0.17
**Vital signs**				
Heart rate (BPM)	−5.83 ± 1.24	−0.67 ± 2.67	−8.53 ± 1.35*	−1.25 ± 2.68
Systolic BP mm Hg	−11.00 ± 1.89	−2.92 ± 4.86	−20.65 ± 2.88***	−5.33 ± 2.55
Diastolic BP mm Hg	−4.53 ± 2.51	0.33 ± 3.54	−11.07 ±± 1.97	−5.42 ± 2.72
**Biochemical parameters**				
Glucose mg/dl	−3.91 ± 2.78	1.91 ± 2.10	−3.33 ± 3.00	1.91 ± 2.68
Triglycerides mg/dl	−6.17 ± 17.43	−3.83 ± 5.20	−7.17 ± 10.31	−1.45 ± 6.10
Total Cholesterol mg/dl	−25.67 ± 4.36	−26.73 ± 8.05	−33.67 ± 6.29	−27.09 ± 5.61
HDL mg/dl	−1.12 ± 0.54	−1.27 ± 0.81	−1.13 ± 1.03	−1.45 ± 0.79
LDL mg/dl	−19.47 ± 3.27	−24.36 ± 7.71	−32.40 ± 5.07	−25.30 ± 5.41
**B) OBESE**	**Changes in the first month**		**Changes in two months**	
	**LC-HS (N** = **10)**	**Control (N** = **10)**	**LC-HS (N** = **10)**	**Control (N** = **10)**
**Anthropometric parameters**				
Body weight	−2.14 ± 0.68	−2.13 ± 0.64	−4.68 ± 0.67*	−2.17 ± 0.95
Body mass index (BMI) kg/m^2^	−0.85 ± 0.30	−0.84 ± 0.23	−1.85 ± 0.34	−0.84 ± 0.34
AC1 (cm)	−3.45 ± 1.15	−1.53 ± 0.45	−6.18 ± 1.16	−3.30 ± 1.49
AC2 (cm)	−3.10 ± 0.86	−1.80 ± 0.74	−5.80 ± 1.16	−5.70 ± 1.79
Triceps skinfold thickness (mm)	−1.71 ± 0.92	−1.68 ± 0.86	−3.29 ± 0.78	−2.38 ± 1.18
% Body fat (BF)	−0.69 ± 0.16	−0.49 ± 0.10	−1.30 ± 0.29	−0.81 ± 0.22
**Vital signs**				
Heart rate (BPM)	−4.05 ± 1.10	−0.50 ± 3.61	−8.00 ± 1.14*	−1.75 ± 2.09
Systolic BP mm Hg	−11.42 ± 2.46	−10.50 ± 7.46	−18.42 ± 3.95*	−13.50 ± 7.11
Diastolic BP mm Hg	−8.40 ± 2.61	−6.00 ± 4.38	−10.70 ± 1.51	−9.88 ± 6.97
**Biochemical parameters**				
Glucose mg/dl	−3.40 ± 4.82	−2.38 ± 2.03	−4.96 ± 3.00	−5.75 ± 1.65
Triglycerides mg/dl	−38.29 ± 27.44	−10.63 ± 17.58	−29.10 ± 26.86	9.37 ± 17.73
Total Cholesterol mg/dl	−15.60 ± 4.41	−19.25 ± 7.90	−20.57 ± 4.53	−14.00 ± 10.66
HDL mg/dl	−1.73 ± 0.62	−3.12 ± 1.89	−2.36 ± 0.73	−2.50 ± 1.88
LDL mg/dl	−15.70 ± 3.38	−15.88 ± 6.59	−25.50 ± 3.44	−14.50 ± 8.91

Intergroup (vs control) statistical analysis is reported as follows: *p < 0.05; **p < 0.01; ***p < 0.001. Data are ex*p*ressed as the mean ± se.

#### Heart rate and blood pressure

There were no significant differences in baseline heart rate and blood pressures parameters between the LC-HS and control groups of overweight and obese volunteers (Table 1). First, a significant decrease was observed in the heart rate of the volunteers of the overweight and obese groups consuming LC-HS after 30 and 60 days, while the heart rate remained unchanged during the entire study period in the control groups (Table 1). After two months, statistically significant differences were observed for the change in heart rate when the two groups were compared (control: −1.25 ± 2.68 bpm vs. LC-HS: −8.53 ± 1.35 bpm in overweight, *p* < 0.05; Table 2A) (control: −1.75 ± 2.09 bpm vs. LC-HS: −8.00 ± 1.14 bpm in obese, *p* < 0.05; Table 2B). Regarding blood pressure, both systolic and diastolic BP in overweight and obese LC-HS groups also showed a significant reduction after 30 and 60 days compared to the basal value (Table 1), and this reduction was greater for the systolic blood pressure, with values of nearly 130 at the beginning of the treatment that dropped below 110 at the end of the treatment (Table 1). However, in the case of the control group, no significant reduction was observed after 60 days. When control and LC-HS groups were compared, we observed significant differences only in the systolic BP at the end of the study (Table 2). Whereas blood pressure dropped 20.65 ± 2.88 and 18.42 ± 3.95 mm Hg in the LC-HS overweight and obese groups respectively, the same parameter decreased only 5.33 ± 2.55 and 13.50 ± 7.11 mm Hg in the overweight and obese control groups respectively (Table 2).

Reduction in blood pressure was accompanied by an improvement in the circulating lipid profile, showing the most significant changes in the overweight group consuming the supplement (Table 1).

#### Adverse events

Treatment of overweight and obese subjects with LC-HS was well-tolerated. The incidence of a reported diarrhea case in the treated group was caused by a viral infection. No subjects had adverse side effects during the intervention period. Accordingly, safety hematological reference values were normal throughout the intervention study (data not shown).

#### Appetite assessment

Although appetite assessment was not strictly evaluated, monitoring of the volunteers throughout the study by a simple survey indicated that most individuals of the LC-HS group experienced a satiating effect, confirming previous observations^18^.

### Hypertrophied adipocytes model results

Recently, the capacity of LC-HS to activate AMPK in the liver of HFD-induced obesity animal model has been reported^17^. Despite the limitations of a cellular model and recognizing the inherent restrictions using high doses and non-metabolized polyphenols, LC-HS combination was assessed for triglyceride accumulation and AMPK activation in a cell model of adipocyte hypertrophy in the context of insulin resistance. The results of this examination are presented below.

#### LC-HS polyphenols decrease intracellular triglycerides in hypertrophied adipocytes

**Figure 2:**
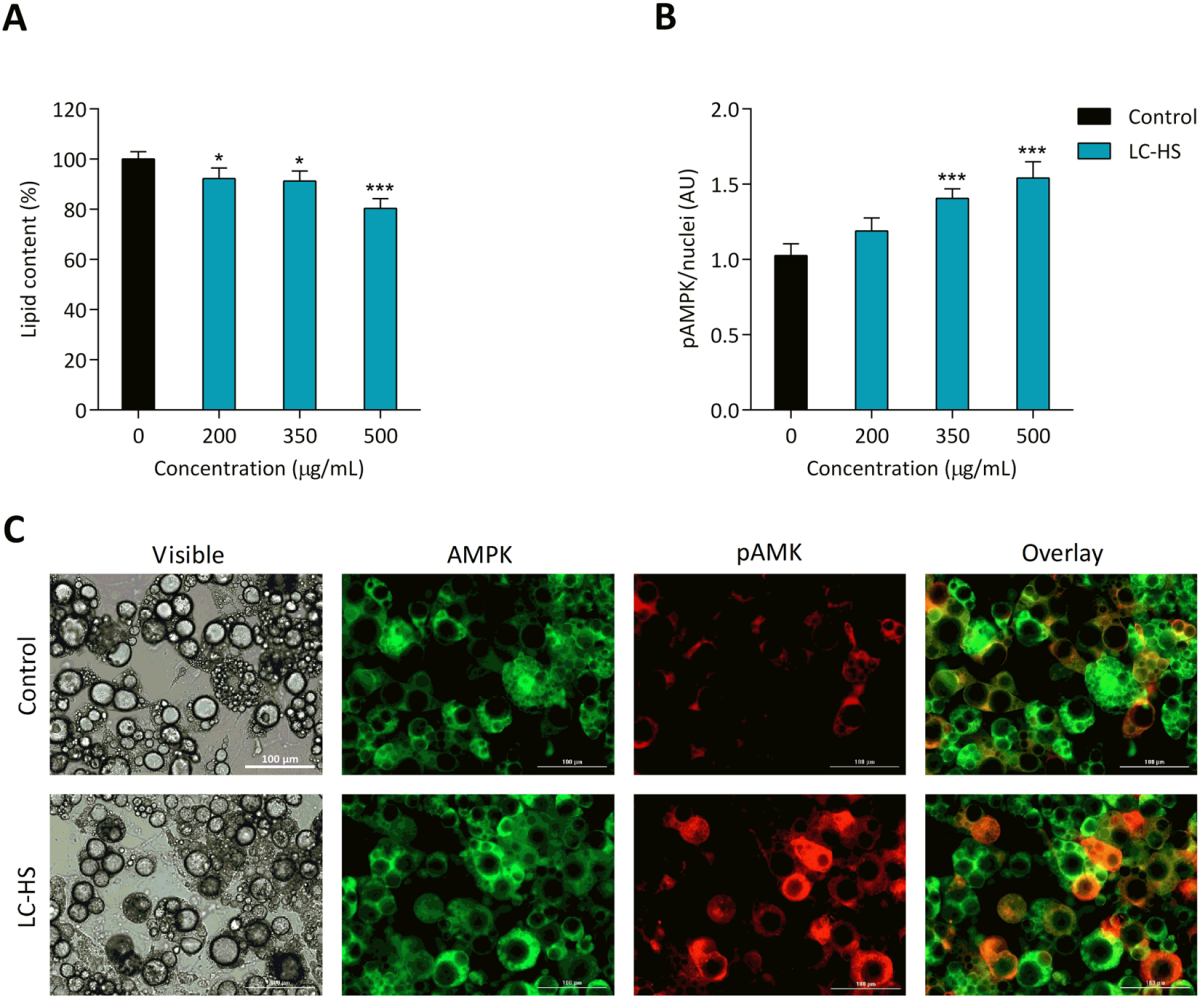
LC-HS combination decreases triglyceride accumulation and activates AMPK in hypertrophied 3T3-L1 adipocytes. Hypertrophied adipocytes were treated with a combination of the polyphenolic extracts, and triglyceride accumulation was monitored by AdipoRed staining as mentioned in the materials section (**A**). Hypertrophied adipocytes were treated with the same concentrations of the polyphenolic combination as in (**A**), and AMPK (non-active) and pAMPK (activated) kinases were quantitated by immunofluorescence microscopy (**B**). Representative photomicrographs of phase contrast, green fluorescence (AMPK), red fluorescence (pAMK) and overlay (**C**). The data are expressed as the mean ± S.D. from three independent experiments performed in octuplicate.

The capacity of the combination of polyphenols derived from *H*. *sabdariffa* and *L*. *citriodora* to decrease triglyceride accumulation was confirmed in a cellular model of hypertrophy. The results showed a dose response behavior in the decrease of the triglyceride accumulation with an increase in the concentration of the polyphenol combination, reaching a reduction of 19.7% at the maximum concentration assayed; i.e., 500 µg/mL (Fig. 2A).

#### Activation of AMPK by LC-HS polyphenols in hypertrophied adipocytes

To determine if the selected combination of polyphenols from LC and HS extracts was able to reduce the lipid content concomitantly with the activation of AMPK, the immunofluorescence detection of AMPK in hypertrophied adipocytes was assayed after treatment with the combination. The polyphenolic extract promoted a potent increase in the pAMPK/nuclei ratio in a dose-dependent manner that was significant compared to the control already at 350 µg/mL and reached an increase approximately 1.5-fold at 500 µg/mL of extract with respect to the control (Fig. 2B). Representative immunofluorescence micrographs of the activated pAMPK (red fluorescence) vs. total AMPK (green fluorescence) are shown in Fig. 2C.

### Characterization of the dietary supplement by High-Performance Liquid Chromatography (HPLC)

**Figure 3:**
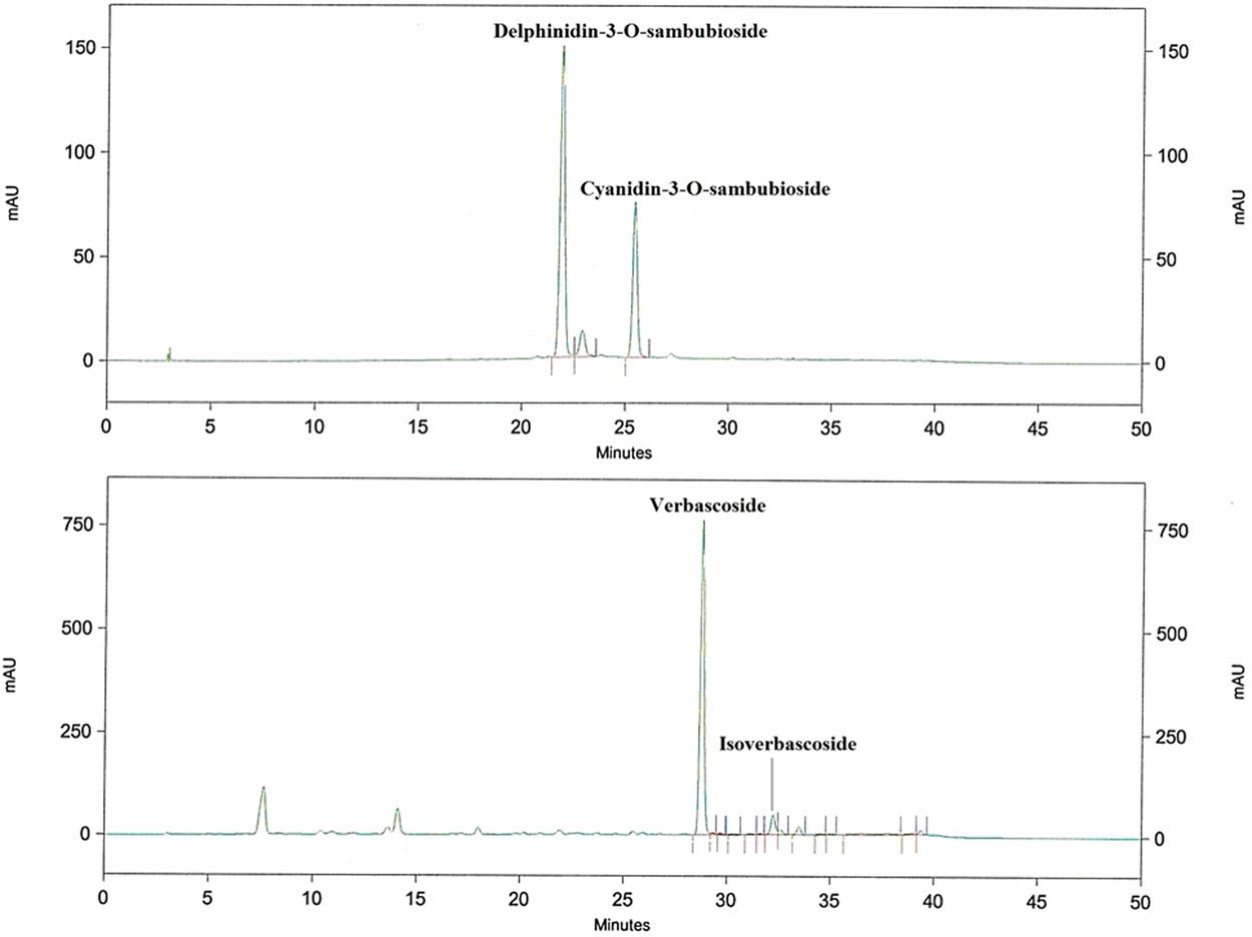
Representative HPLC chromatograms of the LC-HS combination at 320 nm for phenylpropanoids (upper panel) and 520 nm for anthocyanins (lower panel). Four compounds including delphinidin-3-O-sambubioside, cyanidin-3-O-sambubioside, verbascoside and isoverbascoside were determined by HPLC.

The composition of the LC-HS combination was analyzed by HPLC-DAD-ESI-IT-MS. Major compounds identified by their UV spectra and MS/MS data of the peaks belonged to the polyphenolic subfamilies of phenylpropanoids and anthocyanins. Figure 3 shows representative chromatograms of the dietary supplement at 520 nm and 320 nm, upper and lower panel respectively. Four major phenolic compounds were identified, i.e. two anthocyanins, delphinidin-3-O-sambubioside and cyanidin-3-O-sambubioside and two phenylpropanoids, verbascoside, and isoverbascoside. Total anthocyanins represented 3.5% of the total dry weight with delphinidin-3-O-sambubioside constituting 2.27% (65% of total anthocyanidins) and cyanidin-3-O-sambubioside comprising 1.23% (35% of total anthocyanidins). Regarding phenylpropanoids, 16% w/w, verbascoside represented the major compound and constituted 15% (93.75% of total phenylpropanoids) and isoverbascoside represented 1% (6.65% of total phenylpropanoids).

## Discussion

### Trial limitations

The limitations of the study may be related with the metabolic variability of the participants since some of them were pre- or post-menopausal women and this may play a role in adiposity and lipid management. However, a similar percentage of women ranged from 45 to 55 years was present in all groups and none of them reported taking hormone replacement therapy. Additional limitations corresponded to the low number of participants and the short time for intervention. Nevertheless, significant differences were observed in anthropometric and cardiovascular parameters when the two groups were compared at the end of the study.

### Applicability of trial findings

Female participants in this study were recruited from a Nutrition and Dietetics Centre in a city of the Southeast of Spain. Then, we can state that the results are applicable to women in urban environments with similar life styles and eating habits. Last, since the results may also vary depending on the composition of the dietary supplement, the results of the present study would be only valid for the specific polyphenolic composition of the herbal extracts studied here.

### Discussion of results

Obesity is defined as a condition of excessive accumulation of fat in adipose tissue that carries a health risk and predisposes to insulin resistance, hypertension and dyslipidemia. Despite the adverse effects of obesity, weight loss can result in a significant reduction in the risk of most of these comorbid diseases^19^. Restricting caloric intake and increasing caloric expenditure via physical activity seem to be the only accepted ways to prevent development of obesity-associated disorders. Nevertheless, in many cases, these changes in lifestyle are not possible, being necessary to implement complementary therapeutical tools, such as bariatric surgery or the intake of dietary supplements containing polyphenolic extracts^20^.

In this last context, we recently reported the capacities of LC polyphenolic extract and its major compound verbascoside to ameliorate high glucose-induced metabolic disturbances. These effects were mediated by the PPARγ-dependent transcriptional upregulation of adiponectin, the mRNA expression upregulation of PPAR-α and downregulation of FASN, in correlation to a potent activation of AMPK^16^. Experiments in a hyperlipidemic mice model suggested a significant improvement in fat metabolism (cholesterol and triglycerides), especially in the triglyceride clearance, but the glucose tolerance test was not affected.

In addition, HS polyphenols have been demonstrated to be particularly effective in decreasing inflammation linked to metabolic stress by the inhibition of leptin and MCP-1 secretions^3,9,14^. The polyphenolic HS extract also prevented hepatic steatosis in hyperlipidemic mice through the modulation of the expression of genes involved in glucose and lipid homeostasis. In animals fed a high fat diet, HS polyphenols attenuated the increase in blood glucose levels and the apparent increase in insulin resistance, as well as increased the respiratory quotients, which are closely related to the basal metabolic rate (BMR). A decrease in the expression of lipogenic genes, such as fatty acid synthase (FASN) and the sterol regulatory element-binding protein (Srebp-1c), concomitantly to the activation of hepatic AMPK, was also reported in this animal model^10^. AMPK activation is known to induce peroxisome proliferator-activated receptor γ coactivator 1α (PGC-1α) expression and directly enhances its activity through phosphorylation, which increases mitochondrial biogenesis and function^21^. These results note that the putative mechanism of HS polyphenols may be mediated by modulation of fat utilization, most likely by the inhibition of lipogenesis and activation of lipolysis, probably through enhancing mitochondrial biogenesis. All these events resemble a metabolic situation that reflects high cellular energy demand and improved energy expenditure and probably a higher BMR.

Taking into account the common and complementary molecular targets reached by LC and HS polyphenols, we strongly considered the possibility to combine both extracts to test their role in the management of obesity-related disorders. First, previous *in vitro* and animal trials led us to establish the dose response behavior and the range of effective polyphenol concentrations. In the present work, the optimum proportion of LC and HS extracts was established based on the estimated daily intake of polyphenols derived from our previous human intervention studies using the extracts in a separate manner^11,14,22–24^.

In spite of both control and experimental groups were taking an isocaloric diet, the results obtained in this study showed that the consumption of 500 mg per day of LC-HS for two months in overweight women significantly reduced body weight, abdominal circumference and percentage of body fat compared to the control group. A similar tendency was observed in obese volunteers consuming the polyphenolic extract, but results were in general less significant, indicating that the supplement was more effective when obesity changes had not been stablished.

Previous results from our laboratory indicate that control of appetite may be one of the candidate mechanisms by which LC-HS polyphenols may act^18^. The satiating effect was not strictly evaluated in the present report but monitoring of the volunteers revealed that LC-HS exerted a certain satiating effect. Our previous studies showed that HS extract reduced leptin levels in hypertrophic adipocytes^9^. In addition, the LC-HS polyphenolic extract increased anorexigenic (glucagon-like peptide -1) and decreased orexigenic (ghrelin) hormones. Aside from satiety, the same study opens the possibility that the extract could act in other body systems, such as the cardiovascular system and the adipose tissue, that have been addressed in the present report.

Regarding cardiovascular system, the consumption of LC-HS significantly decreased heart rate and systolic blood pressure compared to the control group, particularly in overweight participants. Traditionally, calyxes from *Hibiscus sabdariffa* have been used to treat hypertension, which is a risk factor of cardiovascular diseases. Animal and human studies have shown that consumption of HS extract reduces systolic and diastolic blood pressure^11,25^. Targeted training or endurance exercise promotes skeletal muscle to adapt to higher mitochondrial density and quality, increased oxygen availability and blood flow, as well as enlarged arteries^26^, which turns to a lower resting heart rate and blood pressure and increased BMR. In a similar way, we hypothesize that LC-HS polyphenols may mimic the effects of endurance exercise on heart rate and blood pressure. In addition, an elevated resting heart rate is also one of the main predictors of cardiovascular mortality and it is associated with sudden cardiac death^27^. Natural agents able to reduce heart rate might become an important therapy in the prevention of sudden cardiac death. A reduction of heart rate has been observed after omega-3 fatty acids supplementation, and one of the mechanisms proposed is a direct effect on cardiac cell membrane electrical excitability^27^. However, the mechanism through which LC-HS reduces heart rate in overweight women is still unknown and deserves further attention. Finally, the selected participants presented moderate dyslipidemia (Table 1). Healthy obese people often exhibit this clinical phenotype^28^. Circulating LDL-cholesterol levels decrease in both groups, suggesting that the change to an equilibrated isocaloric diet could be the main responsible factor for this observation.

In the context of the adipose tissue, a previous report revealed that the decrease in body fat mass in the LC-HS group correlated with a reduction in circulating leptin levels compared to the control^18^. Our first interpretation was based on the lipostatic role that leptin plays, otherwise said, the reduction in body fat mass may be accompanied by a decrease in leptin secretion by adipocytes, taking into account that we could not assess the presence of leptin resistance in participants. Since leptin is a hormone secreted by adipocytes, these results strongly suggest a direct action of LC-HS extract on adipocytes. This prompted us to gain more inside by preliminary exploring the possible molecular mechanisms through the use on the *in vitro* adipocyte model. Although cellular systems are limited for studying human diseases, the insulin resistant hypertrophied 3T3-L1 adipocytes induced by a high glucose concentration used in this study and others from our group, resemble the metabolic alterations associated with excessive food storage in adipose tissue of obese subjects^4,29–31^ and provides a valid model to explore candidate molecular targets of LC-HS polyphenols^32,33^.

Among the putative molecular targets, AMPK is proposed as one of the candidates to consider due to the previous results on the potential molecular complementary effects of HS and LC on lipid and glucose metabolism and the reported activation of AMPK by both polyphenolic extracts *in vitro*^9,16^ and in hyperlipidemic mice^10,16,17^. The LC-HS polyphenolic extract resulted in a strong AMPK activation of approximately 1.5-fold compared with that of the control at the highest concentration utilized in hypertrophied 3T3-L1 adipocytes. AMPK activation has been shown to increase mitochondrial biogenesis and density, which in turns should result in an increase in the maximum metabolic rate (MMR), the basal metabolic rate (BMR), and exercise tolerance^34^. In fact, increased voluntary activity has been observed in mice artificially selected for a high BMR^35^, whereas mice with impaired AMPK function had decreased voluntary activity^36^, which suggests a direct link between the cellular energy state, BMR, and voluntary activity that is likely partly mediated by AMPK. The results obtained in obese rats using a synthetic estrogen have shown an increase in resting energy expenditure, through increased heat production and oxygen consumption and a reduced respiratory quotient in correlation with an induction of liver AMPK phosphorylation with a fatty acid utilization increase^37^. AMPK also enhances the metabolic rate by activating phosphofructokinase-2 (PFK2), a key bifunctional enzyme that regulates the rates of glycolysis and gluconeogenesis^38^, by mimicking, in this way, starvation or low glucose conditions. Therefore, we hypothesize that AMPK activation by polyphenols may increase BMR and energy expenditure, leading to improved metabolic and anthropometric parameters, as reported for some alternative weight loss agents capable of increasing the basal metabolism and oxygen consumption^37,39,40^. Conceivably, dietary therapies with bioactive compounds potentially acting on protein kinase AMPK, such as some polyphenols, may have potential benefits in the amelioration of obesity-related diseases.

Interestingly, it is known that activation of AMPK in the hypothalamus enhances food intake and its inhibition by leptin decreases appetite^41^. It may be suggested that our polyphenolic extract might normalize leptin levels in adipose tissue, inhibiting AMPK in the hypothalamus and suppressing feeding behavior. Altogether, the satiating^18^ and blood pressure normalization effects (this report) observed suggest that hypothalamus could be a key target organ for the action of the extract together with adipose tissue. Nevertheless, all these hypotheses require further investigation in the future by using animal models. On the basis of the potential activity observed for polyphenols in adipose tissue and liver, efforts to provide data on randomized control trials, as is the case of this study, deserve further consideration in the future.

In conclusion, our results show that the intake of LC-HS polyphenols for two months in overweight women decreased weight, improved anthropometric parameters, decreased systolic BP and heart beat and improved the blood lipid profile. However, changes were more modest in obese volunteers. Probably, more drastic changes in lifestyle or longer treatments should be carried out by obese subjects to achieve more consistent results. Therefore, the consumption of 500 mg/day of LC-HS, in combination with an isocaloric diet, may be considered as a dietary intervention for weight management and the prevention of metabolic syndrome, being AMPK one of the candidate molecular targets to explore in future research.

## Materials and Methods

### Chemicals and reagents

Dulbecco’s modified Eagle’s medium (DMEM), Dulbecco’s phosphate buffered saline (PBS) and penicillin-streptomycin were purchased from Gibco (Grand Island, NY, USA). Dexamethasone (DEX), 3-isobutyl-1-methylxanthine (IBMX) and insulin were obtained from Sigma-Aldrich (Madrid, Spain). Calf serum (CS) and fetal bovine serum (FBS) were purchased from ThermoFisher Scientific (Cramlington, Northumberland, UK). Cellulose acetate filters (0.2 µm) were obtained from GE Healthcare Life Sciences (Buckinghamshire, UK). *Hibiscus sabdariffa* polyphenolic extract (10% anthocyanins, dry weight), *Lippia citriodora* polyphenolic extract (25% verbascoside, dry weight) and the combination of both extracts selected to be tested in the clinical trial (MetabolAid®) were kindly provided by Monteloeder, SL (Elche, Alicante, Spain).

### Characterization of the dietary supplement by HPLC-DAD-ESI-IT-MS

The combination of polyphenolic extracts (LC-HS) was prepared by mixing LC and HS extracts at a weight ratio (w/w) of 65:35. MetabolAid® was provided by Monteloeder S.L. (Alicante, Spain) (Patent application number P201731147). The composition of the combination of extracts was identified and quantified by using an HPLC instrument (Agilent LC 214 1100 series; Agilent Technologies, Inc., Palo Alto, CA, USA) controlled by Chemstation software, as previously reported^9,42^. The HPLC instrument was coupled to an Esquire 3000 + (Bruker Daltonics, GmbH, Bremen, Germany) mass spectrometer equipped with an ESI source and ion trap mass analyzer and controlled by Esquire control and data analysis software. A Merck Lichrospher 100RP-18 (5 μm, 250 × 4 mm) column was used for analytical purposes. The main compounds were identified by HPLC-DAD analysis, comparing the retention time, UV spectra, and MS/MS data of the peaks in the samples with those of authentic standards or data reported in the literature.

### Maintenance of the 3T3-L1 cell line and differentiation to hypertrophied adipocytes

The 3T3-L1 preadipocytes (American Type Culture Collection, Manassas, VA, USA) were cultured in low glucose (1 g/L) DMEM supplemented with 10% CS, 100 µg/mL streptomycin and 100 U/mL penicillin and incubated at 37 °C in a humidified (5% CO_2_, 95% air) atmosphere. Differentiation from preadipocytes to adipocytes was induced by high glucose (4.5 g/L) DMEM supplemented with 10% FBS, 1 µM insulin, 1 µM DEX and 0.5 mM IBMX for 48 h. Then, cells were maintained in high glucose DMEM with FBS and insulin, and the medium was replaced every 2 days obtaining hypertrophied adipocytes after 20 days of incubation, a well-established insulin resistant adipocyte model bearing metabolic stress^4,16^. Once hypertrophied adipocytes were obtained, cells were treated with LC-HS extract at several concentrations for 72 h. For cell treatment, the extract was dissolved in medium and filtered for sterilization.

### Evaluation of lipid content by AdipoRed

Lipid content of hypertrophied adipocytes was assessed using AdipoRed^TM^ Reagent (Lonza, Walkersville, MD, USA). Briefly, supernatant was removed from the cells, and the cells were then washed carefully with PBS. Next, AdipoRed was added and incubated for 15 minutes at room temperature. Triglyceride accumulation was measured using a fluorescence microplate reader (POLARstar, Omega, BMG LABTECH) at 485 nm excitation and 572 nm emission.

### Determination of activation of AMPK by immunofluorescence

To study the activation of AMPK in hypertrophied adipocytes, AMPK phosphorylated in Thr172 (pAMPK) was quantified with an immunofluorescence assay. Cells were fixed with a fixation buffer (Cytofix^TM^, BD Biosciences, Europe), permeabilized with 0.3% Triton X-100 (Sigma-Aldrich, Spain) and blocked with 4% goat serum (Sigma-Aldrich). Then, cells were incubated overnight at 4 °C with mouse monoclonal AMPK alpha 1 + AMPK alpha 2 antibodies (Abcam, Cambridge, UK) and rabbit monoclonal phospho-AMPKα (Thr172; Cell Signalling Technology, Danvers, MA, USA). After incubation with primary antibodies, cells were washed with PBS and incubated for 6 h at room temperature together with each corresponding polyclonal secondary antibody, goat anti-rabbit IgG CF™ 594 and anti-mouse-FITC, all from Sigma-Aldrich (St. Louis, MO, USA). Fluorescence from cells was measured using a cell imaging multi-mode microplate reader (Cytation 3, Biotek, Spain) at 593 nm excitation and 614 nm emission for pAMPK levels and 490 nm excitation and 520 nm emission for AMPK levels. Activation of AMPK was expressed as levels of AMPK normalized to total AMPK (pAMPK/AMPK). Microphotographs of pAMPK and AMPK were taken at 20x.

### Study design and study population

The study was an 8-week, randomized, double-blind, placebo-controlled trial. The study participants were recruited during 2015 from the city of Elche, Alicante, Spain. Before participation in the study, subjects were informed by the investigators about the purpose and the study procedures. All subjects provided written informed consent (ICF) before entering the study and the Ethical Committee of the Miguel Hernández University of Elche approved the study protocol (reference IB.ER.01.15). The study was conducted in accord with the Helsinki Declaration (1983 version). During the enrollment period, 82 prospective overweight volunteers were recruited at the pharmacy in March 2015.

Exclusion criteria included total cholesterol lower than 200 mg/dL, presence of any obesity-related pathology, use of prescription medication for cholesterol or hypertension, hormone replacement therapy, consumption of antioxidant supplements/drugs, alcohol addiction and women who were pregnant or lactating.

Based on the above criteria, fifty-five healthy women, aged 36–69 years old, with a body mass index (BMI) from 24 to 34 kg/m^2^ passed a telephone-based health screening and interview as well as the biochemical and anthropometrical evaluation. After recruitment, the subjects were randomly assigned into the control (N = 26) or experimental group (N = 29) in a 1:1 ratio for BMI using an Excel program by the research team. Participants (identified by a code) and researchers responsible for the follow-up were blinded. During the study (April-July 2015), 9 volunteers dropped out, 6 in the control and 3 in the intervention group, and a total of 46 volunteers completed the study (control, N = 20 and intervention, N = 26) (Fig. 1).

The control group (mean age 51) received two capsules of placebo (400 mg of crystalline microcellulose each), and the treatment group (mean age 52) received two capsules, each one containing 250 mg of LC-HS and 150 mg excipients (crystalline microcellulose). Composition of capsules was established based in previous studies performed in humans^11,14,22,24^. The capsules were made to have the same size, same odor and same color (red capsule), both for LC-HS and placebo. After overnight fasting, volunteers were instructed to take two capsules 20–30 minutes prior to breakfast every day for two months. During the screening visit, demographic and lifestyle information was collected (age, dietary and physical habits, alcohol consumption and smoking habits).

Women were instructed by a qualified dietician to follow an isocaloric and balanced diet of 2,200 kcal. At baseline, all participants completed a validated semiquantitative food frequency questionnaire^43^. The questionnaire included 22 items, among others, grains, pulses, meat, eggs, seafood, dairy products, fruits, vegetables, processed foods, snacks high in fat and sugar and beverages. For each item, participants could select from 3 frequency categories (daily, weekly or monthly) and the number of servings from each food category. Besides, throughout both the pre and post-intervention survey, a 15-min interview, adapted from a validated questionnaire was conducted with each participant to gather information for a 24 h diet recall^43^. Participants in both groups (dietary supplement and placebo) were given personalized advice for dietary changes at achieving a diet as close as possible to isocaloric and balanced diet with normal hydration. During the meeting, women were also asked about daily physical activity. All declared to be absolutely sedentary. Therefore, they were advised walking for at least half an hour per day. Trained dieticians were responsible for all aspects of the intervention. Compliance of the subjects with the ingestion of capsules, diet and exercise was assessed at each visit or by phone every week during the 2 months of study, estimated as a reasonable time to observe anthropometric changes in participants^44^. Measurements were taken before and after 30 and 60 days of the study. Before analysis (Tables 1 and 2), data were stratified in four groups: overweight and obese control, and overweight and obese volunteers consuming the polyphenolic extract.

### Efficacy outcomes

The efficacy outcome measurements were taken at baseline and after 30 and 60 days of the intervention period. Moreover, during each visit, symptoms or side effects were recorded.

The main objective of the study was to assess the effect of taking LC-HS extracts on changes in anthropometric and circulating parameters in volunteers. To this end, the following parameters were assessed. Anthropometric measurements included body weight, height, triceps skinfold thickness and abdominal circumference (AC) measured at two different sites: anteriorly midway between the xiphoid process of the sternum and the umbilicus and laterally between the lower end of the rib cage and iliac crests (AC1) and at umbilicus level (AC2). A scale with a height measuring rod was used for the measurement of body weight and height. Body mass index (BMI) was derived from body weight and height using the equation BMI = body weight (kg)/height^2^ (m). Triceps skinfold thickness was measured using a skinfold caliper, and AC1 and AC2 were measured using a tape measure. Percentage of body fat (% BF) was calculated from the weight, height and abdominal circumference (AC1 and AC2) using the Weltman equation for obese women^41^. Fasting blood was collected to determine total glucose and glycosylated hemoglobin (HbA1c) and the lipid profile, which included triglycerides, total cholesterol, high-density lipoprotein (HDL) and low-density lipoprotein (LDL)-cholesterol. Blood was also analyzed for safety parameters, hematology, electrolytes (Na, K), creatinine, urea, uric acid, glutamic-pyruvic transaminase (GPT), glutamic-oxaloacetic transaminase (GOT) and C-reactive protein.

Furthermore, the secondary objective of the study was to assess the effect of taking LC-HS extracts on changes in blood pressure in volunteers. To this end, systolic (SBP) and diastolic blood pressure (DBP) and heart beat were measured at rest at the beginning and at 30 and 60 days after the intervention using an Omron HEM-7320-LA oscillometric blood pressure monitor (Omron Healthcare Co. Ltd, Kyoto, Japan) at the upper arm with a large cuff. Five independent measurements for SBP, DBP and heart rate were taken using a validated protocol^45^.

### Theoretical estimation of sample size

Theoretical estimation of sample size was essentially as previously described^18^. This allowed to estimate a minimum sample size of around 17 women per group in order to detect significant changes in body composition.

### Stopping guidelines

Participants compliance and retention was esentially as described previously^18^. The following reasons were considered for the exclusion of the participants after being included in the trial: personal conflict (n = 2 in control and n = 1 in LC-HS group), protocol violations (n = 2 in control), accident (n = 1 in control), medication intake (n = 1 in control), pathology (n = 1 in LC-HS group) and pregnancy (n = 1 in LC-HS group).

### Statistical analysis

Clinical statistical analyses were performed with a Student’s t-test using Graphpad Prism software. Outcome variables were assessed for conformance to the normal distribution and Kolmogorov-Smirnov test was utilized for transformation when needed. Data are reported as the mean ± se (standard error of the mean). Reported *p*-values were two-sided and a P-value of 0.05 or less was considered statistically significant for between-group comparisons. Comparisons were established between the control and the LC-HS groups by unpaired Student’s t-test. By contrast, intra-group statistical analysis at the endpoint was compared to the baseline and analyzed by paired Student’s t-test. Statistically significant differences throughout the study were expressed as *p < 0.05; **p < 0.01, ***p < 0.001.

The results of an AdipoRed assay and immunofluorescence assay were represented using Graphpad Prism software and are expressed as the mean ± standard deviation. The parameters studied were compared to controls and analyzed using a one-way ANOVA and Tukey’s test for multiple comparisons.

## Supplementary information

Full trial protocol
