# Supplementary material for: Differential effects of a combination of Hibiscus sabdariffa and Lippia citriodora polyphenols in overweight/obese subjects: A randomized controlled trial
Source: Sci Rep. 2019 Feb 28;9:2999. doi: 10.1038/s41598-019-39159-5 (PMC6395806; doi:10.1038/s41598-019-39159-5)
Supplement: Supplementary file 1 — Full trial protocol [file 41598_2019_39159_MOESM1_ESM.docx]

**SUPPLEMENTARY INFORMATION**

**CLINICAL TRIAL PROTOCOL**

**Differential effects of a combination of *Hibiscus sabdariffa* and *Lippia citriodora* polyphenols in overweight/obese subjects: A randomized controlled trial**

**Authors:**

María Herranz-López ^1^†, Mariló Olivares-Vicente ^1^†, Marina Boix-Castejón ^2^, Nuria Caturla ^3^, Enrique Roche ^2, 4, ‡^, and Vicente Micol ^1 , 4, ‡,^ *

**Affiliation:**

^1^ Instituto de Investigación, Desarrollo e Innovación en Biotecnología Sanitaria de Elche (IDiBE) e Instituto de Biología Molecular y Celular (IBMC). Miguel Hernández University (UMH), Elche 03202, Alicante, Spain.

^2^ Institute of Bioengineering and Department of Applied Biology-Nutrition, University Miguel Hernandez, Alicante Institute for Health and Biomedical Research (ISABIAL-FISABIO Foundation), Alicante, Spain

^3^ Monteloeder S.L., Alicante, Spain

^4^ CIBER, Fisiopatología de la Obesidad y la Nutrición, CIBERobn, Instituto de Salud Carlos III (CB12/03/30038). Spain

† These authors contributed equally to this work.

‡ These authors shared author co-seniorship.

***Corresponding author:**

Vicente Micol, Tel: 34966658430, Fax: 34966658758 email: [vmicol@umh.es](mailto:vmicol@umh.es)

**Co-authors email:** [mherranz@umh.es](mailto:mherranz@umh.es) (Maria Herranz-Lopez), [maria.olivaresv@umh.es](mailto:maria.olivaresv@umh.es) (Marilo Olivares-Vicente), [marinaboix@hotmail.com](mailto:marinaboix@hotmail.com) (Marina Boix-Castejon), [nuriacaturla@monteloeder.com](mailto:nuriacaturla@monteloeder.com) (Nuria Caturla), [eroche@umh.es](mailto:eroche@umh.es) (Enrique Roche).

**Project summary**

Our previous research in hypertrophied adipocytes and obese animal models has identified *Hibiscus sabdariffa* (HS) and *Lippia citriodora* (LC) with the capacity to activate the AMPK enzyme, decrease metabolic stress in glucotoxicity and/or lipotoxicity events, inhibit triglyceride accumulation, oxidative stress, secretion of inflammatory adipokines, decreased lipogenesis, enhanced fatty acid oxidation, prevention of hepatic steatosis, lowering blood pressure and improving endothelial function. Therefore, a randomized controlled trial was conducted to evaluate if a dietary supplement, containing 500 mg of both *LC* and *HS* extracts (*LC*+*HS*), is capable to normalize anthropometric and circulating parameters in overweight/obese subjects under risk of developing metabolic syndrome. In this trial, 26 women were supplemented with capsules containing 500 mg of *LC*+*HS* for a 2-month period. A placebo group was formed by 20 women consuming capsules containing 400 mg of crystalline microcellulose. The consumption of the dietary supplement for two months showed significant decreases of body weight, abdominal circumference, body fat %, heart rate and systolic blood pressure compared to the placebo. Changes were more prominent in overweight volunteers consuming the polyphenolic extract. The outcomes support that *LC*+*HS* can decrease obesity/overweight-related diseases, probably through the complementary effects between *LC* and *HS* polyphenols.

**General information**

1. **Protocol title**: Evaluation of a dietary supplement for weight control in overweight/obese volunteers (approved by the Ethics Committee of University Miguel Hernandez, Spain).
2. **Protocol code**: NCT03568877 (22/06/2018)
3. **Ethical approval**: University Miguel Hernandez code: IB.ER.01.15 (approval date: March 12^th^, 2015). The official approval letter is attached herewith for reference (Appendix section).
4. **Sponsors:**
5. Spanish Ministry of Economy and Competitiveness (MINECO). Code: AGL2015-67995-C3-1-R.
6. Generalitat Valenciana. Codes: PROMETEO/2016/006, APOSTD/2017/023 and ACIF/2016/230.
7. Instituto de Salud Carlos III, Fisiopatologia de la Obesidad y la Nutricion, CIBERobn. Code: (CB12/03/30038.
8. SME instrument phase 1 European Union funding. Code: INNOPREFAT 683933 (H2020-SMEINST-1-2015_18-03-2015)*.*
9. **Investigators details, address and roles:**
10. Dr Maria Herranz-Lopez

Associated Professor

Institute of Molecular and Cell Biology (IBMC),

University Miguel Hernandez (UMH), Avda de la Universidad sn, 03202-Elche, Alicante, Spain.

Tel: +34966658430

Role: Investigator

1. Marilo Olivares-Vicente

Pre-doctoral fellow

Institute of Molecular and cell Biology (IBMC),

University Miguel Hernandez (UMH), Avda de la Universidad sn, 03202-Elche, Alicante, Spain.

Tel: +34966658430

Role: Investigator

1. Marina Boix-Castejon

Pre-doctoral fellow

Institute of Bioengineering (IB),

University Miguel Hernandez (UMH), Avda de la Universidad sn, 03202-Elche, Alicante, Spain.

Tel: +34965222029

Role: Investigator

1. Dr Nuria Caturla

Company Research Officer

Monteloeder SL,

Elche Industrial Park, Miguel Servet 16, 03203-Elche, Alicante, Spain.

Tel: +34965685275

Role: Company Consultant

1. Dr Enrique Roche

Head of Research,

Institute of Bioengineering (IB)

University Miguel Hernandez (UMH), Avda de la Universidad sn, 03202-Elche, Alicante, Spain.

Tel: +34965222029

Role: Investigator

1. Dr Vicente Micol

Head of Research

Institute of Molecular and Cell Biology (IBMC)

University Miguel Hernandez (UMH), Avda de la Universidad sn, 03202-Elche, Alicante, Spain.

Tel: +34966658430

Role: Main Investigator

1. **Clinical laboratories**:
2. Pharmacy Office (Maria A Iborra-Campos)

Material for determination of anthropometric parameters and blood pressure

101, Carrer Dr Caro

03201-Elche

Alicante, Spain.

Tel: +34965442985

1. Clinical Analysis Laboratory (Jose M Adsuar-Pomares)

Chemicals and devices for blood analysis

7, Carrer Ample

03202-Elche

Alicante, Spain.

Tel: +34965457945

1. **Rationale and background information**

Obesity has reached global epidemic proportions. In addition, obesity is associated with several metabolic disorders including insulin resistance, endothelial dysfunction and dyslipidemia, which are involved in the development of type 2 diabetes, hypertension and cardiovascular diseases. While there has been considerable progress in understanding the molecular mechanisms underlying metabolic disorders, their successful treatment remains limited [1]. Recently, the energy sensor AMP-activated protein kinase (AMPK) has been proposed as an important therapeutic target for obesity therapy that can be modulated by diet and exercise [2]. Our proposal is to investigate the modulation of AMPK by dietary supplements.

Our previous research has accumulated enough evidence and has identified several herbal extracts with the capacity to activate the AMPK enzyme, i.e., *Hibiscus sabdariffa* (*HS*), *Lippia citriodora* (*LC*) and *Olea europaea* leaf and extra virgin olive oil extracts [3-6]. Using an insulin resistant hypertrophic adipocyte model, we have seen that *HS* polyphenols have the capability to decrease metabolic stress in glucotoxicity and/or lipotoxicity events through the modulation of pathways associated with energy management and inflammation [7]. Additionally, HS polyphenols have exhibited the capacity to inhibit triglyceride accumulation, oxidative stress and the secretion of inflammatory adipokines that regulate the infiltration of circulating macrophages to adipose tissue [6]. Moreover, the efficacy of *HS* polyphenolic extract has also been demonstrated in animal models, preventing hepatic steatosis in hyperlipidemic mice through the regulation of the expression of genes involved in glucose and lipid homeostasis, lowering blood pressure and improving endothelial function [8, 9]. In fact, a bioavailability study conducted in rats showed that the major *HS* metabolites found in the plasma of rats were quercetin aglycone and its glucuronide [10].

On the other hand, studies on *LC*, namely lemon verbena, polyphenols showed favorable effects such as decreased lipogenesis, enhanced fatty acid oxidation and activation of the AMPK pathway, probably through PPAR-gamma receptor activation and adiponectin, which are involved in the observed beneﬁcial effects [4]. Similar to the *HS* polyphenol extract, the effects observed for *LC* extract have also been corroborated in animal hyperlipidemic models in which the continuous consumption of the *LC* extract prevented fatty liver disease and improved lipid metabolism. Interestingly, the results on lipid and glucose metabolism obtained in the hyperlipidemic mice revealed the possibility that *HS* and *LC* reach complementary targets [4, 8], which prone us to design a dietary supplement in which both extracts were present.

**References:**

[1] Iyer, A.; Fairlie, D.P.; Prins, J.B.; Hammock, B.D.; Brown, L. Inflammatory lipid mediators in adipocyte function and obesity. *Nat. Rev. Endocrinol.* **6**, 71-82 (2010).

[2] Carling, D. AMPK signalling in health and disease. *Curr. Opin. Cell Biol.* **45**, 31-37 (2017).

[3] Beltran-Debon, R. *et al*. The aqueous extract of *Hibiscus sabdariffa* calices modulates the production of monocyte chemoattractant protein-1 in humans. *Phytomedicine.* **17**, 186-191 (2010).

[4] Herranz-Lopez, M. *et al*. Lemon verbena (*Lippia citriodora*) polyphenols alleviate obesity-related disturbances in hypertrophic adipocytes through ampk-dependent mechanisms. *Phytomedicine.* **22**, 605-614 (2015).

[5] Jimenez-Sanchez, C. *et al*. AMPK modulatory activity of olive–tree leaves phenolic compounds: Bioassay-guided isolation on adipocyte model and in silico approach. *PLoS ONE.* **12**, e0173074 (2017).

[6] Menendez, J.A. *et al.* Xenohormetic and anti-aging activity of secoiridoid polyphenols present in extra virgin olive oil: A new family of gerosuppressant agents. *Cell Cycle.* **12**, 555-578 (2013).

[7] Joven, J. *et al.* Multifunctional targets of dietary polyphenols in disease: A case for the chemokine network and energy metabolism. *Food Chem. Toxicol.* **51**, 267-279 (2013).

[8] Joven, J. *et al.* Plant-derived polyphenols regulate expression of miRNA paralogs miR-103/107 and miR-122 and prevent diet-induced fatty liver disease in hyperlipidemic mice. *Biochim. Biophys. Acta.* **1820**, 894-899 (2012).

[9] Joven, J. *et al.* *Hibiscus sabdariffa* extract lowers blood pressure and improves endothelial function. *Mol. Nutr. Food. Res.* **58**, 1374-1378 (2014).

[10] Fernandez-Arroyo, S. *et al*. Bioavailability study of a polyphenol-enriched extract from *Hibiscus sabdariffa* in rats and associated antioxidant status. *Mol. Nutr. Food. Res.* **56**, 1590-1595 (2012).

**8. Study objectives**

1. To assess the effect of taking LC-HS extracts on changes in anthropometric parameters in overweight/obese volunteers.
2. To assess the effect of taking LC-HS extracts on changes in circulating parameters in overweight/obese volunteers.
3. To assess the effect of taking LC-HS extracts on changes in blood pressure in overweight/obese volunteers.

**9. Study protocol**

**9.1 Study design**

The study was an 8-week, randomized, double-blind, placebo-controlled trial, designed to evaluate the potential of *HS*+*LC* extracts in modulation of anthropometric and circulating parameters as well blood pressure in overweight and obese Spanish women.

82 women, habitual clients of a Pharmacy Office in Elche (Spain), passed a health evaluation protocol. Anthropometric parameters, including weight, height and waste/hip ratio were determined on-site at the Pharmacy Office. Blood pressure determinations were taken with a monitor in the same Pharmacy Office, according to current regulations provided by the European Society of Hypertension. Finally, circulating glucose, cholesterol and triglyceride levels were measured with a Rapid Control Gauge Reflotron^®^ Plus (Roche). To proceed to selection, contacted volunteers passed a telephone health screening interview based in the inclusion and exclusion criteria. Demographic and lifestyle information was collected as well during the telephone interview, including age, dietary and physical habits. Once recruited (N= 55), women were randomly assigned into the control (N = 26) or experimental group (N = 29) in a 1:1 ratio according to BMI (24-34 kg/m^2^) using an Excel program by the research team. The control group (mean age 51) received capsules of placebo (400 mg of crystalline microcellulose each), and the experimental group (mean age 52) received capsules, each one containing 250 mg of *HS*+*LC* plus 150 mg excipients (crystalline microcellulose). The capsules provided to both groups were made to have the same size, same odour and same colour (red capsule).

During the 60-days intervention, volunteers were instructed to take two capsules 20–30 min after overnight fasting and prior to breakfast every day. In addition, women were instructed by a qualified dietician to follow an isocaloric and balanced diet (55% carbohydrates, 33% lipids and 12% proteins) of 2,200 kcal. At baseline, all participants completed a validated semiquantitative food frequency questionnaire. The questionnaire included 22 items to determine habitual consumption of grains, pulses, meat, eggs, seafood, dairy products, fruits, vegetables, processed foods, snacks high in fat and sugar and beverages. For each item, participants could select from 3 frequency categories (daily, weekly or monthly) and the number of servings from each food category. Besides, throughout both the pre and post-intervention survey, a 15-min interview, adapted from a validated questionnaire was conducted with each participant to gather information for a 24 h diet recall. Participants in both groups (dietary supplement and placebo) were given personalized advice for dietary changes at achieving a diet as close as possible to isocaloric and balanced diet with normal hydration. During the meeting, women were also asked about daily physical activity and were advised to walk for at least 30 minutes per day. Trained dieticians were responsible for all aspects of the intervention. Compliance of the subjects with the ingestion of capsules, diet and exercise was assessed at each clinic visit or by phone every week during the 2 months of study.

Measurements were taken at the beginning (day 0) and at day 30^th^ and 60^th^ of the intervention. Data were analysed and compared to the start of the study and between groups at the same time period of the intervention. These comprised anthropometric determinations, circulating parameters and blood pressure values. Anthropometric measurements included body weight, height, triceps skinfold thickness and abdominal circumference (AC) measured at two different sites: anteriorly midway between the xiphoid process of the sternum and the umbilicus and laterally between the lower end of the rib cage and iliac crests (AC1) and at umbilicus level (AC2). Body weight and height were measured using a scale with a height measuring rod. Body mass index (BMI) was derived from body weight and height using the equation BMI = body weight (kg) / height^2^ (m). Triceps skinfold thickness was measured using a skinfold caliper, and AC1 and AC2 were measured using a tape measure. Percentage of body fat (% BF) was calculated from the weight, height and abdominal circumference (AC1 and AC2) using the Weltman equation for obese women.

Regarding circulating parameters, fasting blood was collected to determine total glucose and glycosylated hemoglobin (HbA1c) and the lipid profile, which included triglycerides, total cholesterol, high-density lipoprotein (HDL) and low-density lipoprotein (LDL)-cholesterol. Blood was also analysed for safety parameters, hematology, electrolytes (Na, K), creatinine, urea, uric acid, glutamic-pyruvic transaminase (GPT), glutamic-oxaloacetic transaminase (GOT) and C-reactive protein.

Finally, systolic (SBP) and diastolic blood pressure (DBP) and heart beats were measured at rest, using an Omron HEM-7320-LA oscillometric blood pressure monitor (Omron Healthcare Co. Ltd, Kyoto, Japan) at the upper arm with a large cuff. Five independent measurements for SBP, DBP and heart rate were taken using a validated protocol. The study flow diagram is shown in Figure 1.


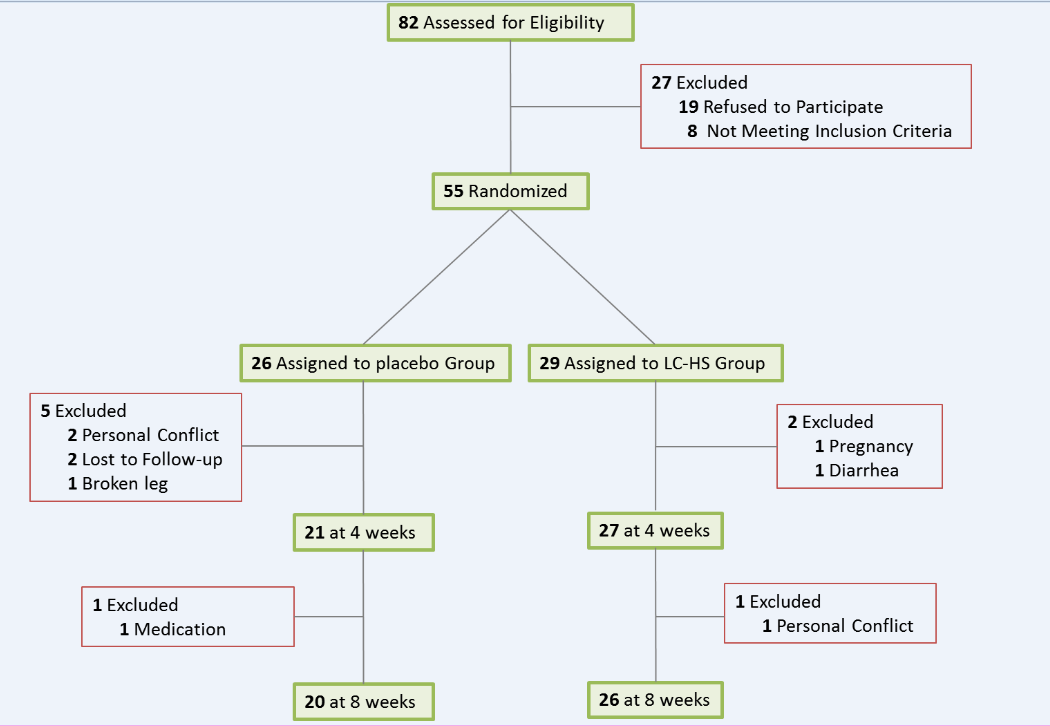


**Figure 1:** Study flow chart.

**9.1.2 Recruitment of volunteers**

55 volunteers were recruited from 82 initially contacted women at the Pharmacy Office in Elche (Spain). Volunteers were selected according to several inclusion and exclusion criteria, as follows:

**9.1.2.1 Inclusion criteria**

1. Written informed consent obtained from volunteers after detailed information provided by investigators about the purpose and procedures of the study.
2. Volunteers age between 36 and 69 years.
3. Diastolic blood pressure > 75 mmHg, Systolic blood pressure > 120 mmHg
4. Women.
5. Having a good record of compliance.

**9.1.2.2 Exclusion criteria**

1. Total cholesterol < 200 mg/dL.
2. Evident presence of an obesity related pathology.
3. Use of medication to treat hypercholesterolemia and/or hypertension.
4. Hormone replacement therapy.
5. Consumption of antioxidant supplements, including vitamins and herbal extracts.
6. Consumption of alcohol and/or drugs.
7. Smoking habits.
8. Women in pregnancy or in lactation periods.

**9.1.2.3 Rights of volunteers to drop out of the study**

Volunteers were free to drop out from the study at any time for any reason without giving explanations. Volunteers may also be dropped out from the study for the following reasons: pathology diagnosis, prescription of medication, accident, protocol violations and at any time at discretion of the investigator. The drop out document must be provided to participants at the same time that the written consent at the beginning of the study.

**9.2 Methodology**

**9.2.1 Blood sampling**

Fasting blood sample were drawn from the volunteers by experienced and well-trained health professionals. 5 mL of blood were transferred into an EDTA (ethylenediamine tetra acetic acid) tube and processed for analysis according to laboratory standard protocols.

**9.2.2 Measured blood parameters:**

**- Circulating cells and related parameters*:**

- Red blood cell counts.

- Hematocrit, mean corpuscular volume, mean corpuscular hemoglobin, mean corpuscular hemoglobin concentration and red blood cell distribution width.

- Leucocyte counts: Neutrophils, lymphocytes, monocytes, eosinophils and basophils.

- Platelet counts.

**- Glycemia profiles**:** Glucose and glycosylated hemoglobin (HbA1C).

**- Lipid profile**:** Triglycerides, total cholesterol, LDL-cholesterol and HDL-cholesterol.

**- Metabolites for kidney function*:** Creatinine, urea and uric acid.

**- Liver and muscle markers*:** Glutamic-pyruvic transaminase (GPT) and glutamic-oxaloacetic transaminase (GOT).

**- Electrolytes*:** Na^+^ and K^+^.

**- Stress markers*:** C-reactive protein (CRP).

(*) Safety parameters to control the health status of participants.

(**) Parameters analyzed in the study.

**9.2.3 Statistical method**

For analysis, data were stratified in four groups: overweight and obese placebo, and overweight and obese volunteers consuming the polyphenolic extract. Clinical statistical analyses were performed with a Student’s t-test using Graphpad Prism software. Outcome variables were assessed for conformance to the normal distribution and transformed if necessary by a KS-test. Data are reported as the mean ± SEM. Reported *p*-values were two-sided and a *p*-value of 0.05 or less was considered statistically significant for between-group comparisons. Comparisons were established between the placebo and the LC-HS groups by unpaired Student’s t-test. By contrast, intra-group statistical analysis at the endpoint was compared to the baseline and analyzed by paired Student’s t-test. Statistically significant differences throughout the study were expressed as **p* <0.05; ** *p* <0.01, *** *p* <0.001.
